# The Use of CSF Multiplex PCR Panel in Patients with Viral Uveitis

**DOI:** 10.3390/diagnostics16010143

**Published:** 2026-01-01

**Authors:** Young Hwan Jeong, Su Hwan Park, Seung Min Lee, Iksoo Byon, Jongyoun Yi, Sung-Who Park

**Affiliations:** 1 Department of Ophthalmology, School of Medicine, Pusan National University, Busan 49241, Republic of Korea; mowhan2@gmail.com (Y.H.J.); isbyon@naver.com (I.B.); 2 Biomedical Research Institute, Pusan National University Hospital, Busan 49241, Republic of Korea; 3 Department of Ophthalmology, Pusan National University Yangsan Hospital, Yangsan 50612, Republic of Korea; sleet12@naver.com (S.H.P.); platinummetal@hanmail.net (S.M.L.); 4 Research Institute for Convergence of Biomedical Science and Technology, Pusan National University Yangsan Hospital, Yangsan 50612, Republic of Korea; 5 Department of Laboratory Medicine, School of Medicine, Pusan National University, Busan 49241, Republic of Korea; socioliberal@pusan.ac.kr

**Keywords:** anterior uveitis, acute retinal necrosis, cytomegalovirus retinitis, herpesvirus, multiplex polymerase chain reaction

## Abstract

**Background/Objectives**: Polymerase chain reaction (PCR) testing of ocular fluids is an essential diagnostic method for identifying infectious causes of uveitis. However, multiplex PCR kits specifically developed for ophthalmic use are not commercially available in many regions, including Korea. Given the biochemical similarity between cerebrospinal fluid (CSF) and aqueous humor, this study evaluated the diagnostic utility of a commercially available CSF multiplex PCR panel for detecting herpesviruses in patients with suspected viral uveitis. **Methods**: We retrospectively reviewed the medical records of patients whose aqueous humor samples were analyzed using a multiplex PCR assay originally designed for CSF testing (Seeplex Meningitis-V1 ACE Detection kit, Seegene, Seoul, Republic of Korea). The samples were obtained between May 2019 and June 2023 at two tertiary referral hospitals. The assay targeted herpes simplex virus types 1 and 2 (HSV-1, HSV-2), varicella-zoster virus (VZV), cytomegalovirus (CMV), Epstein–Barr virus (EBV), and human herpesvirus 6 (HHV-6). Patients were classified into three groups: (I) anterior uveitis with suspected herpesviral infection, (II) acute retinal necrosis (ARN), and (III) CMV retinitis. Baseline characteristics, PCR positivity rates, and virus prevalence were compared among the groups. **Results**: Among 149 eyes tested, 86 were included in the final analysis. The overall positivity rate was 38.4%. PCR positivity was 19.7% (12/61) in Group I, 93.8% (15/16) in Group II, and 66.7% (6/9) in Group III. CMV was the most common pathogen in Groups I (66.7%) and III (100%), while VZV was predominant in Group II (80%). No HHV-6 infection was detected. **Conclusions**: The positivity rate in anterior uveitis (Group I) was lower than previously reported, likely due to the limited sample volume relative to the assay’s requirement. Nevertheless, the assay demonstrated diagnostic reliability comparable to previous reports for ARN and CMV retinitis. Therefore, the CSF-based multiplex PCR panel serves as a feasible and cost-effective diagnostic option for sight-threatening posterior segment infections, facilitating prompt diagnosis and treatment, although further optimization is warranted for anterior uveitis.

## 1. Introduction

Uveitis, a potentially blinding inflammatory disease, affects approximately 200 individuals per 100,000 in Western populations [1] and up to 714 per 100,000 in developing countries [2]. This condition carries a significant burden, with more than half of patients experiencing complications and up to 35% developing severe visual impairment [3]. Given the contrasting treatment approaches, differentiating infectious uveitis from noninfectious types, such as autoimmune uveitis, is crucial.

Various diagnostic techniques have been applied to identify infectious organisms in uveitis. Polymerase chain reaction (PCR) for detecting deoxyribonucleic acid (DNA) of Toxoplasma gondii was first introduced in ophthalmology in 1990 [4] and has since been extended to detect nucleic acids from other pathogens such as herpesviruses, rubella virus, and Mycobacterium tuberculosis. PCR has been widely utilized in uveitis because of its rapid and reliable identification of causative pathogens [5]. Several studies have demonstrated the diagnostic usefulness of PCR using aqueous humor [6,7,8,9], with high sensitivity for detecting minute quantities of pathogenic DNA or ribonucleic acid (RNA) [10]. This molecular approach has proven valuable for the diagnosis of viral anterior uveitis and viral retinitis in both immunocompetent and immunocompromised individuals [4].

In several regions—including Japan, Europe, and the United States—ophthalmic-adapted multiplex or broad-range PCR systems have been developed or implemented [11]. These assays, which range from multiplex viral panels to strip-based PCR formats, have demonstrated high diagnostic sensitivity and specificity in infectious uveitis and are optimized for the very small sample volumes typically obtained from aqueous or vitreous taps [12]. Nonetheless, such platforms remain unstandardized globally and are largely limited to specialized centers, restricting their accessibility and widespread clinical use.

In Korea, commercially available multiplex PCR kits specifically designed for ophthalmic samples are not yet accessible, limiting their routine application in clinical practice. This gap highlights the need for alternative diagnostic approaches in settings where ophthalmic-specific multiplex assays are unavailable.

In contrast, multiplex PCR assays designed for cerebrospinal fluid (CSF) analysis have been commercially developed and are widely used [13,14,15]. These CSF panels can detect major herpesviruses such as herpes simplex virus types 1 and 2 (HSV-1, HSV-2), varicella-zoster virus (VZV), Epstein–Barr virus (EBV), cytomegalovirus (CMV), and human herpesvirus 6 (HHV-6). Given the biochemical and structural similarities between CSF and aqueous humor [16,17], we presumed that a CSF multiplex PCR assay could also be applied effectively to ocular samples for the detection of viral pathogens.

Therefore, this study aimed to evaluate the diagnostic utility of a commercially available CSF multiplex PCR assay for detecting herpesviruses in patients with uveitis suspected of viral infection. By analyzing positivity rates and viral prevalence across different uveitis subtypes, we sought to determine whether the CSF-based multiplex PCR panel could serve as a feasible and cost-effective alternative for diagnosing viral uveitis in clinical practice.

## 2. Materials and Methods

### 2.1. Study Population

This retrospective study included uveitis patients suspected of having a viral etiology who underwent multiplex PCR testing using a CSF multiplex PCR panel between May 2019 and June 2023 at two tertiary referral hospitals (Department of Ophthalmology at Pusan National University Hospital and Pusan National University Yangsan Hospital). Patients were excluded if they had (1) follow-up periods too short to confirm their clinical diagnosis or (2) diagnoses later revised to other disease entities such as HLA-B27-associated uveitis, noninfectious uveitis, syphilis, toxoplasmosis, toxocariasis, lymphoma, endophthalmitis, or bacterial/fungal infections. As this retrospective study analyzed anonymized medical records, the requirement for written informed consent was waived by the Institutional Review Board of Pusan National University Hospital (IRB No. 2507-010-153).

### 2.2. Diagnostic Procedures

All patients underwent comprehensive ophthalmic examinations, including slit-lamp examination, wide-field fundus photography (Optos California^®^, Optos PLC, Dunfermline, UK), and swept-source optical coherence tomography (SS-OCT; DRI OCT-1 Triton, Topcon, Tokyo, Japan). Systemic evaluations included complete blood count, liver function tests, renal function tests, urinalysis, and serologic testing for syphilis, tuberculosis, toxoplasmosis, and toxocariasis, as well as genetic testing for HLA-B27 and HLA-B51.

Aqueous humor sampling was performed immediately when herpetic etiology was first suspected and before initiation of antiviral therapy. Approximately 0.05–0.1 mL of aqueous humor was aspirated via anterior chamber paracentesis after topical anesthesia and betadine disinfection using a 30-gauge needle under sterile conditions. In patients with bilateral uveitis, aqueous humor was collected from the eye with more severe inflammation. The testing was outsourced to a specialized commercial laboratory (Seegene Medical Foundation, Seoul, Republic of Korea). Multiplex PCR was performed on the aqueous humor samples using the Seeplex Meningitis-V1 ACE Detection kit (version 2.0; Seegene, Seoul, Republic of Korea) according to the manufacturer’s instructions [18].

Final diagnoses were made by specialists based on clinical features, disease course, and treatment response, in conjunction with PCR results. Diagnoses were not determined in a masked manner. PCR findings also informed subsequent patient management: all patients with positive PCR results were promptly started on appropriate oral antiviral therapy according to the identified pathogen, whereas in PCR-negative cases, the decision to initiate antiviral treatment was individualized based on the clinician’s assessment of the clinical features, degree of inflammation, and treatment response. PCR results additionally assisted clinicians in adjusting corticosteroid regimens and refining the final diagnosis.

### 2.3. Patient Classification

Patients were classified into three groups based on diagnoses made by retinal specialists according to the Standardization of Uveitis Nomenclature (SUN) criteria [19], taking into account slit-lamp, fundus, and OCT findings before and after PCR testing. Group I included patients with anterior uveitis in whom herpesviral infection was suspected based on elevated intraocular pressure (IOP) before steroid treatment, inadequate inflammatory control with corticosteroids, or characteristic findings such as sectoral iris depigmentation or keratic precipitates (KPs) [20,21,22]. Group II comprised patients diagnosed with acute retinal necrosis (ARN) according to the criteria established by the American Uveitis Society [23]. Group III included patients with CMV retinitis who met all of the following criteria: an immunocompromised condition, ill-defined retinal necrosis lesions, and characteristic fundus findings such as wedge-shaped, hemorrhagic, or granular retinitis [24,25,26].

### 2.4. Statistical Analysis

Baseline characteristics, PCR positivity rates, and the distribution of detected pathogens were compared among the three groups. Statistical analyses were performed using SPSS software (SPSSWIN ver. 22.0; IBM Corp., Armonk, NY, USA). Group comparisons were conducted using one-way analysis of variance (ANOVA) for continuous variables and chi-square tests for categorical variables, followed by Bonferroni-adjusted post hoc pairwise testing when appropriate. Exact *p*-values were reported unless *p* < 0.0001.

For PCR positivity rates in Groups I–III, 95% confidence intervals (95% CIs) were calculated using binomial proportions. In Group I, additional comparisons between PCR-positive and PCR-negative subgroups were performed using independent t-tests and chi-square tests. A *p*-value < 0.05 was considered statistically significant. Continuous variables were expressed as mean ± standard deviation (SD).

## 3. Results

### 3.1. Demographics and Clinical Characteristics

Multiplex PCR test was performed on 149 eyes. Of these, 11 eyes with short follow-up and 52 eyes later re-diagnosed as other diseases (e.g., HLA-B27-associated uveitis, syphilis, lymphoma, endophthalmitis, etc.) were excluded.

Eighty-six patients (86 eyes) were included in the final analysis. The mean age was 56.6 ± 17.0 years; 55 (64.0%) were male and 31 (36.0%) were female.

Group allocation was as follows: 61 anterior uveitis (Group I), 16 ARN (Group II), and 9 CMV retinitis (Group III).

Group-wise mean ages were 54.0 ± 17.2 (I), 63.4 ± 16.7 (II), and 62.1 ± 13.3 (III) with *p* = 0.085; sex distribution differed among groups (M:F = 44:17, 3:13, 8:1; *p* < 0.001).

**Figure 1:**
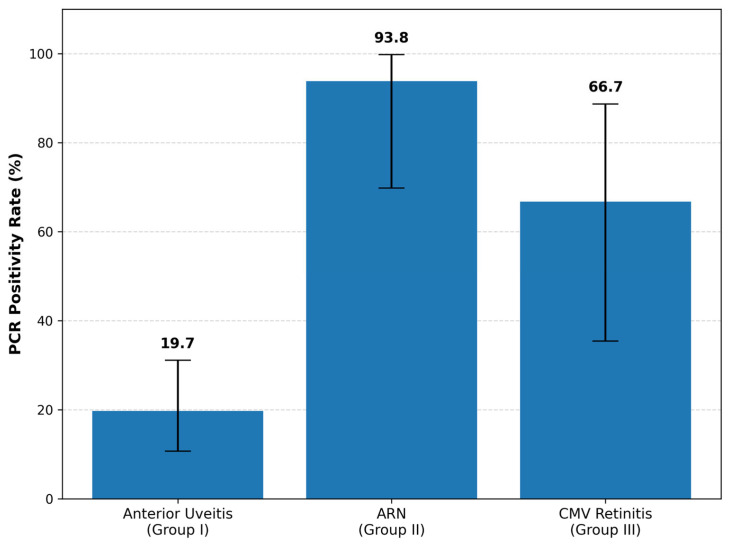
PCR positivity rates across the three uveitis groups. Group I showed a positivity rate of 19.7%, Group II 93.8%, and Group III 66.7%. Error bars represent 95% confidence intervals.

The overall PCR positivity rate was 38.4% (33/86 eyes). Group-specific positivity rates were 19.7% in Group I (12/61; 95% CI, 10.7–31.1%), 93.8% in Group II (15/16; 95% CI, 69.8–99.8%), and 66.7% in Group III (6/9; 95% CI, 35.4–88.7%), showing a statistically significant difference among the groups (*p* < 0.0001), as shown in Figure 1.

Baseline demographics and PCR positivity rates are summarized in Table 1.

### 3.2. Viral Pathogen Distribution

Among PCR-positive eyes, the distribution of detected viral pathogens differed by group (Table 2).

In Group I, CMV was most frequently identified (8/12, 66.7%), followed by VZV (2/12, 16.7%) and HSV-1 (2/12, 16.7%). In Group II, VZV predominated (12/15, 80.0%), followed by HSV-2 (2/15, 13.3%) and CMV (1/15, 6.7%). In Group III, CMV was detected in all cases (6/6, 100%).

The distribution of viral pathogens detected by multiplex PCR among the three groups is summarized in Table 2.

EBV was detected in 3 eyes (2.0%) among the 149 tested eyes but all were excluded after re-evaluation (toxoplasmosis or bacterial keratitis). No HHV-6 infection was detected in any patient.

### 3.3. Subgroup Analysis in Anterior Uveitis (Group I)

In Group I, 55.7% (34/61) of patients had received topical corticosteroids before sampling. However, there were no statistically significant differences in PCR positivity between the steroid-pretreated group and the untreated group (*p* = 0.186). Additionally, no significant differences were observed in age, sex, prevalence of keratic precipitates (KPs), intraocular pressure (IOP), or anti-glaucoma medication history.

Detailed comparisons are presented in Table 3.

## 4. Discussion

Viral uveitis can be destructive and irreversible, emphasizing the importance of prompt diagnosis and treatment [27,28]. However, the diagnosis of infectious uveitis often relies on clinical presentation, which can be ambiguous and challenging, particularly in atypical cases [8]. Although PCR has revolutionized differential diagnosis in uveitis, it still has several limitations, including the invasive nature of aqueous humor sampling, limited sample volume, and the capacity to identify only one pathogen at a time. The introduction of multiplex PCR has addressed many of these challenges by enabling the simultaneous detection of multiple pathogens [9]. Despite its advantages, the lack of commercially available multiplex PCR kits specifically designed for ophthalmic use in Korea has hindered its widespread application.

To overcome this limitation, we investigated the feasibility of repurposing the Seeplex Meningitis-V1 ACE Detection kit, originally developed for CSF analysis, to diagnose herpesviral uveitis. This kit detects HSV-1, HSV-2, VZV, EBV, CMV, and HHV-6 in CSF and has demonstrated high sensitivity and specificity in identifying viral pathogens in patients with acute meningitis [29]. Given the compositional similarity between aqueous humor and CSF [16,17], with CSF constituents influencing aqueous humor through microcirculation [30], we hypothesized that the assay would effectively detect viruses in aqueous humor samples. However, this CSF multiplex PCR panel targets only major herpesviruses (HSV-1, HSV-2, VZV, CMV, EBV, HHV-6) and does not include other relevant viral etiologies of uveitis, such as rubella virus, which is associated with Fuchs heterochromic uveitis. Therefore, PCR-negative results should be interpreted with these diagnostic limitations in mind. Therefore, in this study, using four years of experience with the Seeplex Meningitis-V1 ACE Detection kit for detecting herpesviruses in uveitis, we aimed to validate its reliability by dividing patients into three groups: anterior uveitis suspected of herpesviral infection, ARN, and CMV retinitis.

Two studies have reported PCR results in anterior uveitis with suspected viral infection. The first, conducted in Taiwan by Hsiao et al. [31], included 102 uveitic glaucoma patients and used the LightMix kit (TIB Molbiol, Berlin, Germany). They reported a positivity rate of 41.2%, with CMV being the most common virus (64.2%). As their PCR system was not multiplex, four separate commercial kits were required to detect HSV, CMV, EBV, and VZV. The second study, by Yoo et al. [32] in Korea, analyzed 88 eyes from uveitic glaucoma patients using the same Seeplex Meningitis kit as in our study. They reported a positivity rate of 31.8%, with CMV as the most common virus (50%).

In Group I of the current study, the positivity rate was 19.7%, lower than the 41.2% reported by Hsiao et al. [31] and 31.8% by Yoo et al. [32]. However, the prevalence of CMV (66.7%) was similar to previous reports (64.2% and 50%, respectively). We initially hypothesized that the lower positivity rate in Group I might be due to prior topical corticosteroid use reducing viral shedding. However, our subgroup analysis revealed no statistically significant difference in PCR positivity based on steroid use history (*p* = 0.186). This suggests that factors other than anti-inflammatory treatment may be responsible for the lower sensitivity.

A more critical factor appears to be the discrepancy between the assay’s designed sample volume and the actual available aqueous humor volume. The Seeplex^®^ kit is optimized for CSF sample volumes of 0.2–1.0 mL. In contrast, the volume of aqueous humor obtained was only 0.05–0.1 mL due to safety concerns during paracentesis.

While this limited volume did not hinder detection in ARN (93.8%) and CMV retinitis (66.7%)—conditions characterized by high intraocular viral loads due to active replication—it may be insufficient for detecting the comparatively lower viral loads typical of anterior uveitis. Therefore, the inherent limitation of using a CSF-based assay (requiring larger volumes) on small aqueous samples likely contributed to the lower sensitivity observed in anterior uveitis.

No significant differences were observed in mean age, sex ratio, keratic precipitates, intraocular pressure, or use of anti-glaucoma medications between PCR-positive and PCR-negative patients in Group I (Table 3). Unlike our study, previous research has reported an association between keratic precipitates and PCR positivity [33].

Group II included clinically diagnosed ARN patients, with a PCR positivity rate of 93.8% (15/16). VZV was detected in 12 patients, HSV-2 in 2, and CMV in 1. Group III included CMV retinitis patients, with a positivity rate of 66.7%; CMV was detected in all positive cases. These rates and pathogen distributions are consistent with previous studies. Harper et al. [34] reported positivity rates of 88.4% in ARN and 82.0% in CMV retinitis using commercial real-time PCR, while Scheepers et al. [28] found rates of 71.4% and 85.7%, respectively, using in-house PCR. Both studies identified VZV as the predominant virus in ARN and CMV in CMV retinitis, consistent with our results. This supports the reliability of the CSF multiplex PCR kit for diagnosing ARN and CMV retinitis.

Given that ARN and CMV retinitis typically present with markedly high intraocular viral loads due to active replication within the retina and choroid, PCR testing of aqueous humor is known to achieve very high sensitivity in these conditions. The ability of the CSF-based multiplex PCR panel to reproduce these high detection rates in our cohort indicates that the assay retains adequate analytical sensitivity even when applied to small aqueous samples. Thus, their high positivity rates serve as a practical benchmark confirming that the multiplex panel performs comparably to established ophthalmic PCR methods.

In addition to these major pathogens, EBV was detected in three cases (2.0%) among the 149 eyes tested, but none were clinically relevant. In none of these cases was EBV suspected as the primary pathogen. The first and third cases were clinically diagnosed as ocular toxoplasmosis based on positive serum Toxoplasma IgM or PCR findings, while the second case was presumed to have bacterial keratitis. The first case initially improved with anti-toxoplasmosis treatment but later experienced recurrent uveitis despite stable serologic findings, suggesting reactivation of toxoplasmosis rather than EBV infection. The second case showed severe intraocular inflammation with corneal ulceration; EBV was detected, but inflammation resolved with topical antibiotics. The third case, referred from another hospital for uncontrolled uveitis, had positive EBV PCR in vitreous and aqueous samples, but clinical features were consistent with toxoplasmosis, and treatment with anti-toxoplasmosis agents was continued. Based on these findings, EBV was not considered the primary causative pathogen in any of the cases. EBV is one of the most prevalent viruses worldwide, infecting more than 90% of the population [35,36]. However, the presence of EBV DNA does not necessarily indicate pathogenicity. Previous studies have reported detection of EBV DNA in ocular fluids without clear clinical relevance [36,37,38].

In contrast to EBV, which was occasionally detected without clinical significance, HHV-6 was not identified in any of our cases. This absence is consistent with previous reports showing that HHV-6–associated ocular infection is exceedingly rare and that intraocular HHV-6 DNA is detected in <2% of ocular fluid samples [39]. In addition, HHV-6 has been reported to be detectable in intraocular tissues without representing true pathogenic infection [40], suggesting that its absence in our cohort reflects the inherently low intraocular prevalence rather than insufficient analytic sensitivity.

In CSF-based validation studies, the Seeplex^®^ Meningitis-V1 ACE Detection kit demonstrated high analytic sensitivity, with reported limits of detection of 11 genomes/mL for EBV and VZV, 12 genomes/mL for HSV-2 and HHV-6, 16 genomes/mL for CMV, and 38 genomes/mL for HSV-1 [29]. The assay also showed excellent analytic specificity, with no cross-reactivity observed with human genomic DNA or reference microbial strains. Although aqueous humor differs from CSF in volume and protein composition, these performance characteristics support the assay’s technical robustness and are consistent with the reliable detection rates observed in ARN and CMV retinitis in our cohort.

Given these performance characteristics, it is also important to consider how differences between aqueous humor and CSF may influence PCR yield. Aqueous humor differs from cerebrospinal fluid in several physicochemical aspects, including substantially lower sample volume, lower protein concentration, and lower viral burden, all of which may theoretically reduce the analytical sensitivity of PCR assays. Nevertheless, prior comparative studies have shown that aqueous humor and CSF share similar immunologic characteristics under inflammatory conditions; for example, patients with Vogt–Koyanagi–Harada disease demonstrate parallel lymphocytic profiles in both fluids despite these compositional differences [17]. These observations suggest that, although the absolute biochemical composition is not identical, the intraocular inflammatory milieu may still provide sufficient viral genomic material for reliable amplification using multiplex PCR platforms originally designed for CSF.

Taken together, these results demonstrate that the commercial CSF multiplex PCR kit provides reliable detection of ocular herpesviruses with positivity rates comparable to previous studies. From a practical perspective, outsourcing to a specialized commercial laboratory (Seegene Medical Foundation) ensured high accessibility and a consistent 24–48 h turnaround time without requiring in-house optimization. Although the assay relies on conventional PCR with gel electrophoresis—which involves a more complex workflow than closed-system real-time PCR—it was the most viable commercial multiplex option available in Korea at the time, balancing comprehensive pathogen coverage with logistical feasibility and economic viability. Single-target PCR assays typically cost 40,000–60,000 KRW (around 27–40 USD) per pathogen in Korea, whereas the multiplex PCR kit costs approximately 62,000 KRW (around 42 USD) while detecting six herpesviruses simultaneously. This makes the multiplex assay more cost-effective than performing multiple individual PCR tests and could therefore serve as a practical diagnostic alternative in clinical settings.

### Limitations

Despite these strengths, this study has several limitations. First, its retrospective design and the heterogeneous timing of aqueous sampling may have introduced selection and temporal bias. More than half of the anterior uveitis patients had received topical corticosteroids before sampling, which may have reduced intraocular viral load and consequently lowered PCR sensitivity. Second, the amount of aqueous humor available for analysis was inherently limited. In this study, only 0.05–0.1 mL of aqueous humor could be safely obtained, whereas the CSF multiplex PCR kit is designed for CSF sample volumes of 0.2–1.0 mL. Although this discrepancy in input volume theoretically raises concerns regarding reduced analytical sensitivity, the positivity rates observed in our cohort were comparable to those reported in previous ophthalmic PCR studies, suggesting that the limited sample volume may not have substantially impaired the assay’s practical diagnostic performance. Third, no gold-standard comparator, such as sequencing or a validated ophthalmic multiplex PCR platform, was available for direct comparison. Fourth, all data were obtained from two tertiary referral centers in Korea, which may limit the generalizability of the findings to other settings or populations. Future prospective studies with standardized sampling protocols, adequate sample volumes, and direct head-to-head comparisons with ophthalmic-specific multiplex assays are warranted.

## 5. Conclusions

Our study demonstrates the differential utility of a commercially available CSF multiplex PCR kit. It proved to be a reliable and valuable diagnostic alternative for ARN and CMV retinitis. However, it demonstrated inferior positivity rates in anterior uveitis, suggesting that further verification is needed, particularly regarding sample volume optimization.

Despite the limitation in anterior uveitis, the assay’s high accessibility and cost-effectiveness may facilitate prompt diagnosis and treatment for sight-threatening posterior segment infections, which is crucial for preventing the potentially devastating consequences of viral uveitis.

## Figures and Tables

**Table 1:** Demographic characteristics and PCR results of the patients with uveitis.

	Group I	Group II	Group III	*p*
Number of patients (%)	61 (70.1)	16 (18.6)	9 (10.3)	-
Age ^a^ (years)	54.0 ± 17.2	63.4 ± 16.7	62.1 ± 13.3	0.085
Male:Female	44:17	3:13	8:1	<0.001
PCR positive rate	19.7% (12/61)	93.8% (15/16)	66.7% (6/9)	<0.001
Most common pathogen	CMV (66.7%)	VZV (80.0%)	CMV (100.0%)	-

Data are presented as the mean ± SD ^a^ or number of patients (%). Group I, anterior uveitis group; Group II, acute retinal necrosis (ARN) group; Group III, cytomegalovirus (CMV) retinitis group; PCR, polymerase chain reaction; VZV, varicella-zoster virus.

**Table 2:** The prevalence of pathogen type in each uveitis group.

	Group I	Group II	Group III
CMV	8	1	6
HHV-6	0	0	0
VZV	2	12	0
HSV-1	2	0	0
HSV-2	0	2	0
EBV	0	0	0
Diagnostic positivity	12/61 (19.7%)	15/16 (93.8%)	6/9 (66.7%)

Data are presented as the number of patients (%). Group I, anterior uveitis group; Group II, acute retinal necrosis (ARN) group; Group III, cytomegalovirus (CMV) retinitis group; HHV-6, human herpesvirus-6; VZV, varicella-zoster virus; HSV-1, herpes simplex virus-1; HSV-2, herpes simplex virus-2; EBV, Epstein–Barr virus.

**Table 3:** Clinical characteristics of the patients with anterior uveitis (Group I).

	PCR-Positive	PCR-Negative	*p*-Value
Number of patients	12	49	
Age ^a^ (years)	55.8 ± 14.9	53.5 ± 17.7	0.828
Male:Female	10:2	34:15	0.338
Prior topical corticosteroid use	9 (75.0%)	25 (51.0%)	0.186
KPs	9 (75%)	29 (59.2%)	0.315
IOP (mmHg)	32.0 ± 13.5	29.0 ± 13.4	0.457
Anti-glaucoma medication history	10 (83.3%)	38 (77.6%)	0.664

Data are presented as the mean ± SD ^a^ or number of patients (%). A value of *p* < 0.05 was considered as statistically significant. PCR, polymerase chain reaction; KP, keratic precipitates; IOP, intraocular pressure.

## Data Availability

The datasets generated and/or analyzed during the current study are not publicly available due to containing sensitive clinical information, but de-identified data are available from the corresponding author upon reasonable request.

## Abbreviations

The following abbreviations are used in this manuscript:

PCR	polymerase chain reaction
CSF	cerebrospinal fluid
ARN	acute retinal necrosis
CMV	cytomegalovirus
VZV	varicella-zoster virus
DNA	deoxyribonucleic acid
RNA	ribonucleic acid
HSV	herpes simplex virus
EBV	Epstein–Barr virus
HHV	human herpesvirus
SUN	standardization of uveitis nomenclature
KPs	keratic precipitates
SD	standard deviation
